# Complete mitochondrial genome of *Dahlica (Dahlica) ochrostigma* Roh and Byun, 2018 (Lepidoptera: Psychidae)

**DOI:** 10.1080/23802359.2019.1660243

**Published:** 2019-09-05

**Authors:** Seung Jin Roh, Da-Som Kim, Bong-Woo Lee, Bong-Kyu Byun

**Affiliations:** a Division of Forest Biodiversity, Korea National Arboretum, Pocheon, Republic of Korea;; b Department of Biological Science and Biotechnology, Hannam University, Daejeon, Republic of Korea

**Keywords:** Mitochondrial genome, Tineoidea, Psychidae, *Dahlica (Dahlica) ochrostigma*

## Abstract

We, herein, report the complete mitochondrial genome of *Dahlica* (*Dahlica*) *ochrostigma.* This species’ genome has a total length of 15,429 bp (GenBank accession number: MK890245), consisting of 13 protein-coding genes, 22 tRNA genes, two rRNA genes, and an A + T rich control region. The nucleotide composition is 39.1% T, 42.8% A, 11.1% C, and 7.0% G. This is the first report of a complete mitochondrial genome of the subfamily Naryciinae, and this mitogenomic sequence can be used as a reference for phylogenetic studies on the family Psychidae.

A recent phylogenetic analysis placed the family Psychidae in the superfamily Tineoidea, with the families Eriocottidae, Tineidae, Meessiidae, and Dryadaulidae as sister groups (Regier et al. 2015). The genus *Dahlica* belongs to Psychidae, and its type species is *Dahlica larviformis* Enderlein, 1912, described by Enderlein in 1912. A total of 44 species of *Dahlica* have been described worldwide, most of them from the Palaearctic region in Europe (41 species) and Asia (three species) (Arnscheid and Weidlich 2017; Roh et al. 2018). Moreover, the genus *Dahlica* and four of its sister genera—*Siederia* Meier, 1957, *Brevantennia* Sieder, 1953, *Postsolenobia* Meier, 1958, and *Praesolenobia* Sieder, 1955—were reviewed; as a result, these four genera were placed as subgenera of *Dahlica* on the basis of the venation of male hindwings, male forewing scale morphology, presence of an epiphysis in males, structure of reproductive organs, and female antennae.

In the present study, specimens of *Dahlica* (*Dahlica*) *ochrostigma* were collected from Daejeon, South Korea (36.34560556, 127.2897222) in February 2019. These specimens were deposited in the Systematic Entomology Laboratory, Hannam University (SEL/HNU), Daejeon, South Korea (sample number: HNUE-RSJ151). The complete mitochondrial genome of these specimens was sequenced with Illumina Hiseq (Macrogen, Inc., Seoul, Korea). A total of 14,368,758 reads were assembled in Geneious Prime (Kearse et al. 2012), generating a 2,169,682,458-base pair sequence. Gene annotation was performed and circularity was checked using the MITOS2 webserver (Bernt et al. 2013, http://mitos.bioinf.uni-leipzig.de/).

The complete mitochondrial genome sequence of *D.* (*D.*) *ochrostigma* (Genbank accession number MK890245) has a total length of 15,429 bp, consisting of 13 protein-coding genes, 22 tRNA, two rRNA genes, and an AT-rich control region. The mitogenome contains 39.1% T, 42.8% A, 11.1% C, and 7.0% G, besides a high A + T content. All of the protein coding genes have ATN as the start codon except for cox1, which starts with TTG. The A + T-rich region is 411 bp long.

The phylogenetic position of *D.* (*D.*) *ochrostigma* was inferred using amino acid sequences of the 13 PCGs of 11 species from 5 superfamilies, including two species from Psychidae (which were used as ingroups) and six species from three superfamilies (used as sister groups). Among the latter, one species from Tischerioidea (*Astrotischeria* sp.) and one from Nepiculoidea (*Stigmella roborella* Johansson)—whose sequences were obtained from Genbank—were selected as outgroups based on the results of Jeong et al. (2018). The Maximum-likelihood analysis (Figure 1) indicated that *D.* (*D.*) *ochrostigma* is a distinct species within Tineoidea and that Psychidae is sister to Tineidae.

**Figure 1.**
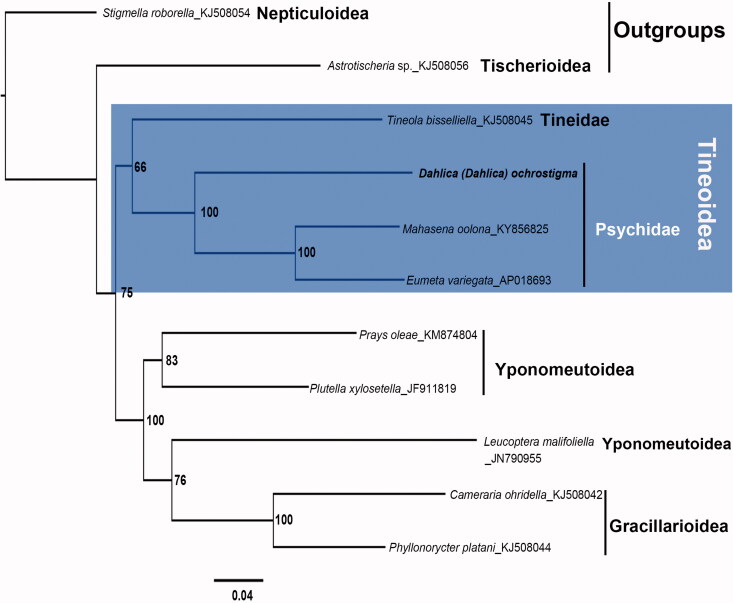
Phylogenetic tree resulting from the maximum-likelihood analysis of concatenated amino acid sequences of 13 mitochondrial protein-coding genes of *D.* (*D.*) *ochrostigma* and other glossatan species. The numbers beside the nodes indicate percentages of 1000 bootstrap values. *Stigmella roborella* (Nepticuloidea) and *Astrotischeria* sp. (Tischerioidea) were used as outgroups. Alphanumeric terms indicate GenBank accession numbers: KJ508054, KJ508056, KJ508045, KY856825, AP018693, KM874804, JF911819, JN790955, KJ508042, and KJ508044.

A support value of 100% was found in the clade formed by *D.* (*D*.) *ochrostigma* and the other Psychidae species. Also, a lower support value was found in the clade formed by Psychidae and Tineidae species. Additional mitogenome sequences are required to infer the precise relationships among groups of Tineoidea.

## Nucleotide sequence accession number

The complete mitochondrial genome sequence of *D.* (*D.*) *ochrostigma* was deposited in GenBank under the accession number MK890245.

## References

[CIT0001] Arnscheid WR, Weidlich M 2017 Microlepidoptera of Europe; Vol. 8 Psychidae. Leiden, Netherlands: Brill.

[CIT0002] Bernt M, Donath A, Juhling F, Externbrink F, Florentz C, Fritzsch G, Putz J, Middendorf M, Stadler P 2013 MITOS: improved de novo metazoan mitochondrial genome annotation. Mol Phylogenet Evol. 69:313–319. 22982435 10.1016/j.ympev.2012.08.023

[CIT0003] Enderlein G 1912 I. Wissenschaftliche Mitteilungen, 2. Zur Kenntnis der Zygophtalmen. Zoologischer Anzeiger. 40:261–282.

[CIT0004] Jeong JS, Kim MJ, Kim SS, Kim I 2018 Complete mitochondrial genome of the female wingless bagworm moth, *Eumeta variegata* Snellen, 1879 (Lepidoptera: Psychidae). Mitochondrial DNA B. 3:1037–1039. 10.1080/23802359.2018.1511851 PMC7799688 33474406

[CIT0005] Kearse M, Moir R, Wilson A, Stones-Havas S, Cheung M, Sturrock S, Buxton S, Cooper A, Markowitz S, Duran C, et al. 2012 Geneious basic: an integrated and extendable desktop software platform for the organization and analysis of sequence data. Bioinformatics. 28:1647–1649. 22543367 10.1093/bioinformatics/bts199 PMC3371832

[CIT0006] Meier H 1957 Ein neues Subgenus und neue Arten aus der Gattung Solenobia Dup. Nachrichtenblatt der Bayerischen Entomologen. 6:55–61.

[CIT0007] Meier H 1958 Der taxonomische Wert der Hinterflügel-Aderung bei den Gattungen Brevantennia Sieder und Solenobia Duponchel (Lep., Psych.). Mitteilungen des naturwissenschaftlichen Vereins für Steiermark. 88:178–192.

[CIT0008] Regier J, Mitter C, Davis DR, Harrison T, Sohn JC, Cummings M, Zwick A, Mitter K 2015 A molecular phylogeny and revised classification for the oldest ditrysian moth lineages (Lepidoptera: Tineoidea): were the megadiverse Ditrysia ancestrally non-phytophagous? Syst Entomol. 40:409–432.

[CIT0009] Roh SJ, Lee BW, Byun BK 2018 Two new species of the genus *Dahlica* Enderlein (Lepidoptera, Psychidae) from Korea. ZK. 733:49–64. 10.3897/zookeys.733.20793 PMC5799782 29416407

[CIT0010] Sieder L 1953 Vorerbeit zu einer Monographie über sie Gattung Solenobia Z. (Lepidopt. Psychidae-Taleporiinae). Zeitschrift der Wiener Entomologischen Gesellschaft. 38:113–128.

[CIT0011] Sieder L 1955 Zweite Vorarbeit über die Gattung Solenobia (Lepidopt., Psychidae, Taleporiinae). Gen. nov. Praesolenobia. Subnet. nov. Solenobia Solenobia Zeller, Spez. nov. *Brevantennia saxatilis*. Zeitschrift der Wiener entomologischen Gesellschaft. 39:241–254.

